# Differential Synaptic Dynamics and Circuit Connectivity of Hippocampal and Thalamic Inputs to the Prefrontal Cortex

**DOI:** 10.1093/texcom/tgaa084

**Published:** 2020-11-06

**Authors:** Sarah Canetta, Eric Teboul, Emma Holt, Scott S Bolkan, Nancy Padilla-Coreano, Joshua A Gordon, Neil L Harrison, Christoph Kellendonk

**Affiliations:** 1 Department of Psychiatry, Columbia University Medical Center, New York, NY 10032, USA; 2 Division of Molecular Therapeutics, New York State Psychiatric Institute, New York, NY 10032, USA; 3 Princeton Neuroscience Institute, Princeton University, Princeton, NJ 08544, USA; 4 Division of Integrative Neuroscience, New York State Psychiatric Institute, New York, NY 10032, USA; 5 Department of Systems Neuroscience, Salk Institute for Biological Studies, La Jolla, CA 92037, USA; 6 National Institute of Mental Health, Bethesda, MD 20892, USA; 7 Department of Molecular Pharmacology and Therapeutics, Columbia University Medical Center, New York, NY 10032, USA; 8 Department of Anesthesiology, Columbia University Medical Center, New York, NY 10032, USA

**Keywords:** interneurons, mediodorsal thalamus, optogenetics, prefrontal cortex, ventral hippocampus

## Abstract

The medial prefrontal cortex (mPFC) integrates inputs from multiple subcortical regions including the mediodorsal nucleus of the thalamus (MD) and the ventral hippocampus (vHPC). How the mPFC differentially processes these inputs is not known. One possibility is that these two inputs target discreet populations of mPFC cells. Alternatively, individual prefrontal cells could receive convergent inputs but distinguish between both inputs based on synaptic differences, such as communication frequency. To address this, we utilized a dual wavelength optogenetic approach to stimulate MD and vHPC inputs onto single, genetically defined mPFC neuronal subtypes. Specifically, we compared the convergence and synaptic dynamics of both inputs onto mPFC pyramidal cells, and parvalbumin (PV)- and vasoactive intestinal peptide (VIP)-expressing interneurons. We found that all individual pyramidal neurons in layer 2/3 of the mPFC receive convergent input from both MD and vHPC. In contrast, PV neurons receive input biased from the MD, while VIP cells receive input biased from the vHPC. Independent of the target, MD inputs transferred information more reliably at higher frequencies (20 Hz) than vHPC inputs. Thus, MD and vHPC projections converge functionally onto mPFC pyramidal cells, but both inputs are distinguished by frequency-dependent synaptic dynamics and preferential engagement of discreet interneuron populations.

## Introduction

The medial prefrontal cortex (mPFC) serves as an integrative hub for both cognitive and affective circuitry. The prelimbic region of the mPFC receives input from multiple subcortical regions including the mediodorsal nucleus of the thalamus (MD) and the ventral aspect of the hippocampus (vHPC) (Parent et al. 2010; Little and Carter 2012; Bolkan et al. 2017). Intriguingly, optogenetic inhibition studies suggest a dissociation in the behavioral function of the two inputs. Inhibition of vHPC to mPFC inputs decreases anxiety and impairs spatial encoding of a working memory task (Spellman et al. 2015; Padilla-Coreano et al. 2016; Abbas et al. 2018). The same manipulation of MD to PFC inputs does not affect anxiety and disrupts maintenance (but not encoding) of spatial information during the same working memory task (Padilla-Coreano et al. 2016; Bolkan et al. 2017). How the PFC differentiates between input coming from these two different subcortical structures is not known.

One possibility is that inputs coming from the vHPC versus MD activate discreet populations of mPFC cells, similar to the concept of “labeled lines” seen in the peripheral sensory system. Optogenetic and electrical stimulation studies suggest pyramidal neurons in layers 2, 3, and 5 may all receive functional inputs from both the vHPC and MD (Floresco and Grace 2003; Parent et al. 2010; Little and Carter 2012; Liu and Carter 2018). However, circuit-mapping studies to date have looked at inputs from the MD and vHPC in isolation, rather than directly testing whether both inputs converge onto the same cells or project to discreet subpopulations.

Alternatively, prefrontal pyramidal cells could distinguish between both inputs based on qualitative differences, such as differences in their frequency-dependent dynamics. Neural oscillations, reflecting the rhythmic fluctuation of neural activity, have been shown to carry behaviorally relevant information. In this context, vHPC–PFC communication relevant to anxiety-related behaviors appears to preferentially occur at lower, theta-range (4–12 Hz) frequencies, while beta-range (20 Hz) frequencies have been shown to be relevant for MD–PFC communication during working memory maintenance (Parnaudeau et al. 2013; Padilla-Coreano et al. 2016; Bolkan et al. 2017). However, these differences in oscillatory dynamics have been examined by correlating bulk temporal activity across the three brain regions during behavior. Such experiments provide minimal insight onto how these structures communicate at the synaptic level.

To address directly the ways in which synaptic inputs from the MD and vHPC differentially target and communicate with prefrontal cells, we utilized optogenetic tools in combination with slice electrophysiology. We focused on neurons in superficial layers 2 and 3 in the prelimbic mPFC because prior work indicated that they are most densely contacted by the MD, while still receiving input from the vHPC (Parent et al. 2010; Little and Carter 2012; Bolkan et al. 2017; Collins et al. 2018). In addition to pyramidal cells, MD and vHPC inputs may be distinguished by their engagement of distinct populations of GABAergic interneurons. We focused on parvalbumin (PV) and vasoactive intestinal peptide (VIP) neurons because they are nonoverlapping populations and display distinct connectivity patterns. Notably, PV interneurons mostly inhibit pyramidal neurons, while VIP interneurons primarily disinhibit them (Fino et al. 2013; Pi et al. 2013; Kubota 2014; Abbas et al. 2018). To test whether MD and vHPC inputs converge onto the same neurons, we implemented a dual-stimulation approach whereby a blue-light sensitive form of Channelrhodopsin2 (Chronos) is injected into the MD, while a red-light sensitive form (ChrimsonR) is injected in the ventral hippocampus (or vice versa) (Klapoetke et al. 2014). Postsynaptic responses to optogenetic stimulation of the two inputs were compared in whole cell patch-clamp recordings obtained from pyramidal neurons and PV and VIP interneurons. To further compare the frequency-dependent synaptic dynamics of MD inputs to those of vHPC inputs, we stimulated them at both 8 and 20 Hz. In aggregate, our data suggest that although inputs from the MD and vHPC converge functionally onto mPFC pyramidal cells, they can be distinguished based on frequency-dependent synaptic dynamics as well as their preferential recruitment of discreet populations of local mPFC interneurons.

## Materials and Methods

### Mice

All animal procedures were approved by New York State Psychiatric Institute’s Animal Care and Use Committee. Parvalbumin Cre (PV-Cre; Jackson Stock #017320) or Vasoactive intestinal peptide Cre (VIP-Cre, Jackson Stock #031628) mice were mated with Ai9 tdTomato reporter mice (Ai9; Jackson Stock # 007909) to produce offspring for ChrimsonR and Chronos dual labeling studies. Adult (6–8 week-old) PV-Cre/Ai9 or VIP-Cre/Ai9 mice were used for viral injections (unilateral injection of either AAV5-Syn-Chronos-GFP or AAV5-Syn-ChrimsonR-tdTomato in the left MD concurrent with unilateral injection of the other virus in the left vHPC) for in vitro optogenetic electrophysiology experiments. Animals were allowed to recover for 8 weeks prior to electrophysiology experiments. Animals were fed ad libitum and reared under normal lighting conditions (12/12 light/dark cycle). Male and female mice were used for all experiments. C57BL/6J (Jackson Labs, Bar Harbor, ME, USA; Stock #000664) or 129SvEvTac (Taconic Biosciences, Germantown, NY; Stock #129Sve) mice were used for single ChannelRhodopsin2 (ChR2) labeling studies. Adult (6–8 week old) C57BL/6J mice were injected with AAV5-hSyn-hChR2 (H134R)-EYFP in the MD and allowed to recover for 6 weeks prior to electrophysiological recordings. Adult (6–8 week old) 129SvEvTac (for 25 °C recordings) or C57BL/6J (for 32 °C recordings) mice were injected with AAV5-CaMKIIa-hChR2(H134R)-mCherry in the vHPC and allowed to recover for 8 weeks prior to electrophysiological recordings.

**Figure 1:**
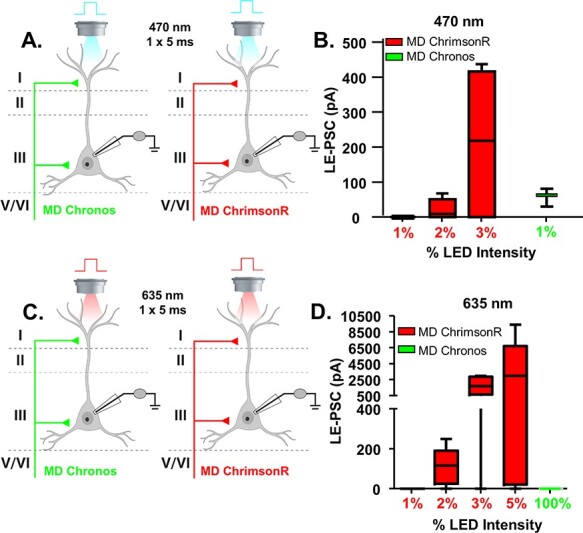
Establishing dual stimulation parameters for activation of Chronos and ChrimsonR. (*A*) Cartoon schematic illustrating the experiment. Animals were injected with either Chronos or ChrimsonR into the MD. Pyramidal cells in layer 2/3 of the mPFC were patched and a single, 5-ms pulse of blue (470 nm) light was delivered via the objective to stimulate neurotransmitter release. (*B*) The average le-PSC response to stimulation of either ChrimsonR or Chronos-containing MD inputs at the indicated light intensities. At 1% of the maximum light intensity, 470 nm light stimulation reliably generated le-PSCs in MD-Chronos mice, while never generating le-PSCs in MD-ChrimsonR mice. (*C*) Cartoon schematic illustrating the experiment. Animals were injected with either Chronos or ChrimsonR into the MD. Pyramidal cells in layer 2/3 of the mPFC were patched and a single, 5-ms pulse of red (635 nm) light was delivered via the objective to stimulate neurotransmitter release. (*D*) The average le-PSC response to stimulation of either ChrimsonR or Chronos-containing MD inputs at the indicated light intensities. Even at 100% of maximum LED intensity, 635 nm light stimulation never generated le-PSCs in MD-Chronos mice.

### Surgery

Sterile stereotactic survival surgery of C57BL/6J, PV-Cre/Ai9 and VIP-Cre/Ai9 mice was performed using the following viral injection volumes and unilateral coordinates: MD (0.3 μL injected at AP −1.2, ML −0.35, DV −3.2 from the skull at bregma) and vHPC (0.2 μL per injection site at the following 10 sites, AP −3 and −3.3, ML +2.8, DV −4.75 from the skull at bregma; AP −3 and −3.3, ML +3.25, DV −4.25 and −2.5 from the skull at bregma; AP −3 and −3.3, ML +3.5, DV −3.9 and −3.3 from the skull at bregma). Virus was injected at the rate of 0.1 μL/min and the pipette was allowed to remain in place between 2 and 5 min at the penetration sites. For the injection of the vHPC in 129SvEvTac mice five injections were done at −3.10 and at −3.30 AP levels for a total of 10 injections in one hemisphere. At each AP level, the five injection sites were −2.90, −4.0; −3.30, −3.60; −3.30, −1.7; −3.70, −3.2; −3.70, −2.5 (ML and DV, respectively). 129SvEvTac coordinates are in mm relative to Bregma (AP, ML) or brain surface at the most medial coordinate (DV) and virus was infused at a rate of 200 nL/min. Viruses were obtained from UNC Vector Core and viral titers are as follows: AAV5-Syn-Chronos-GFP (5 × 10^12^), AAV5-Syn-ChrimsonR-tdTomato (4.2 × 10^12^), AAV5-hSyn-hChR2(H134R)-EYFP (4.8 × 10^12^), AAV5-CaMKIIa-hChR2(H134R)-mCherry (4.5 × 10^12^).

**Figure 2:**
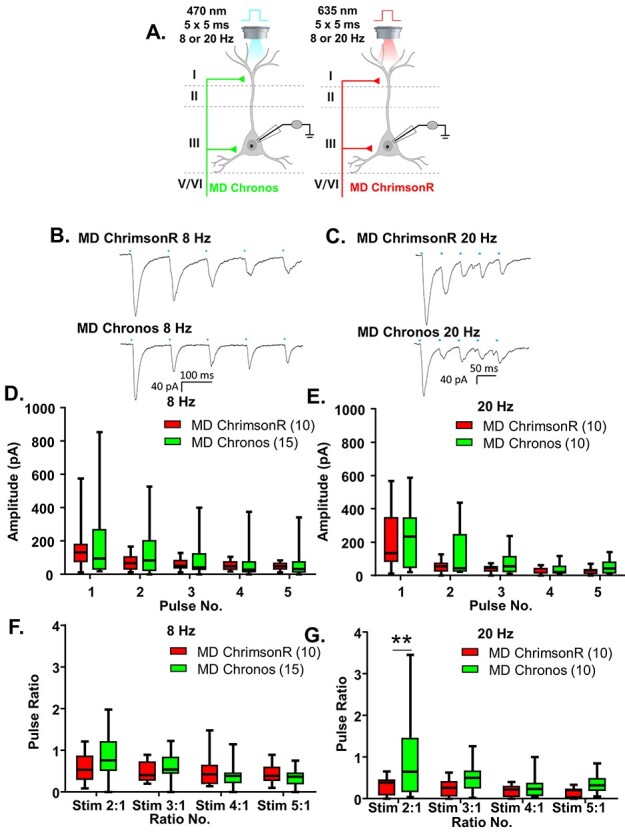
Chronos and ChrimsonR show comparable response properties. (*A*) Cartoon schematic illustrating the experiment. Animals expressing either Chronos or ChrimsonR in the MD were stimulated with five, 5-ms pulses of 470 or 635 nm light, respectively, at a frequency of 8 or 20 Hz. (*B*) Example traces of currents evoked in layer 2/3 mPFC pyramidal cells in response to five, 5-ms pulses of 470 nm light stimulating MD-Chronos or MD-ChrimsonR terminals at 8 Hz. (*C*) Example traces of currents evoked in layer 2/3 mPFC pyramidal cells in response to five, 5-ms pulses of 635 nm light stimulating MD-ChrimsonR or MD-Chronos terminals at 20 Hz. (*D*, *E*) The amplitudes evoked in response to stimulation of either MD-Chronos or MD-ChrimsonR terminals did not differ at 8 Hz (*D*) or 20 Hz (*E*). (*F*) The ratio of responses to repeated light stimuli relative to the first stimulus did not differ between stimulation of MD-Chronos or MD-ChrimsonR terminals at 8 Hz. There were no Bonferroni-corrected posthoc differences between the ChR2 variant for any of the ratios. (*G*) The ratio of responses to repeated light stimuli relative to the first stimulus was slightly different between stimulation of MD-Chronos or MD-ChrimsonR terminals at 20 Hz. Bonferroni-corrected posthoc comparisons of ChR2 variants at each ratio revealed that the ratio of the response to the second stimulus relative to the first was significantly smaller for stimulation of MD-ChrimsonR relative to MD-Chronos terminals at 20 Hz. ^**^P < 0.01.

### Electrophysiology

Whole-cell voltage clamp recordings were performed in visually identified pyramidal cells and genetically identified PV- and VIP-expressing interneurons in the prelimbic (PrL) region of layer 2/3 of the mPFC. Recordings were obtained with a Multiclamp 700B amplifier (Molecular Devices, Sunnyvale, CA, USA) and digitized using a Digidata 1440A acquisition system (Molecular Devices) with Clampex 10 (Molecular Devices) and analyzed with pClamp 10 (Molecular Devices). Following decapitation, 300 μm slices containing mPFC were incubated in artificial cerebral spinal fluid containing (in mM) 126 NaCl, 2.5 KCl, 2.0 MgCl_2_, 1.25 NaH_2_PO_4_, 2.0 CaCl_2_, 26.2 NaHCO_3_ and 10.0 D-glucose, bubbled with oxygen, at 32 °C for 30 min before being returned to room temperature for at least 30 min prior to use. During recording, slices were perfused in artificial cerebral spinal fluid (with drugs added as detailed below) at a rate of 5 mL/min. A recording temperature of 25 °C was used for all experiments except for one subset of experiments in mice expressing ChR2(H134R) in the MD or vHPC, which was performed at 32 °C. Unless specifically indicated in the figure, recording temperatures were always 25 °C. Electrodes were pulled from 1.5 mm borosilicate-glass pipettes on a P-97 puller (Sutter Instruments). Electrode resistance was typically 3–5 MΩ when filled with internal solution consisting of (in mm): 130 CsOH monohydrate, 130 D-Gluconic acid (50%), 10 HEPES, 2 MgCl_2_, 0.2 EGTA, 2.5 MgATP and 0.3 NaGTP (pH 7.3, 280 mOsm; used for ChrimsonR and Chronos dual injection recordings) or 130 K-gluconate, 5 NaCl, 10 HEPES, 0.5 EGTA, 2 MgATP and 0.3 NaGTP (pH 7.3, 280 mOsm; used for ChR2(H134R) single injection recordings).

Pyramidal cells were visually identified based on their shape and prominent apical dendrite at 40x magnification under infrared and diffusion interference contrast microscopy using an Olympus BX51W1 microscope coupled to a Hamamatsu C8484 camera (Olympus America). The recorded cell was placed in the center of the field of view, held at −70 mV in voltage clamp and the current response evoked by a four- or five-pulse train of 5 or 1-ms pulses of blue light (470 nm) or red light (635 nm) delivered at 8 or 20 Hz by a light-emitting diode (LED; CoolLED Olympus America) was recorded. For single ChR2(H134R) stimulation experiments, the LED intensity was varied between 5 and 20% of maximum intensity to produce detectable light-evoked postsynaptic currents (le-PSCs) of a comparable size, when possible. For dual ChrimsonR and Chronos stimulation experiments, the 470 nm LED intensity was kept at 1% and the 635 nm LED intensity was varied between 2 and 90% to produce detectable le-PSCs of a comparable size, when possible. Each experiment consisted of a four or five pulse train of 5 or 1-ms pulses of blue or red light delivered at 8 or 20 Hz; this stimulation paradigm was repeated 5 times for a given experiment and the current traces generated from all five stimulations were averaged and filtered at 2000 Hz with an eight-pole low-pass Bessel filter. Although minimal stimulation intensities were used, occasionally light stimulation provoked an action potential-like current instead of a le-PSC (identified based on response shape as well as amplitude). For the percent responding analyses, we included these large-amplitude responses in our averages. For the response ratio analyses, these large amplitude responses were excluded from the averages. In two instances, this resulted in the need to exclude a cell from the response ratio analysis that was included in the percent responding analysis, because all stimulus repetitions contained an action-potential like response. Le-PSCs from the averaged current traces were manually identified in MiniAnalysis (Synaptosoft). The relevant parameters for each response (event onset time, event peak time, maximum current amplitude) were imported into Matlab where they could be sorted based on experimental condition.

For a subset of experiments, 1 μM tetrodotoxin (TTX) in combination with 200 μM 4-aminopuridine (4-AP) was added to the bath is isolate monosynaptic transmission. We did not include TTX and 4-AP in all experiments because TTX/4-AP decreases the amplitude of the evoked postsynaptic response, and therefore biases towards not detecting a connection when one might in fact exist.

### Immunostaining

Proper targeting of the MD and vHPC was verified for all mice used for electrophysiology. After mPFC slices were prepared, the remaining brain tissue was postfixed by immersion fixation in 4% paraformaldehyde at 4° for at least 48 h. The tissue was then washed for 24 h in phosphate buffered saline (PBS) before 100 μm slices through the MD and vHPC were prepared on a vibratome (Leica). Immunostaining to detect EYFP, GFP, or mCherry was performed using the following primary antibodies: EYFP or GFP (chicken-anti-GFP; Abcam; ab13970, 1:1000), mCherry (rabbit-anti-dsRed; Takara Bio; 632 496, 1:500); tdTomato was visible without additional amplification. Alexa Fluor-conjugated secondary antibodies (Invitrogen, 1:1000) were used for secondary detection.

**Figure 3:**
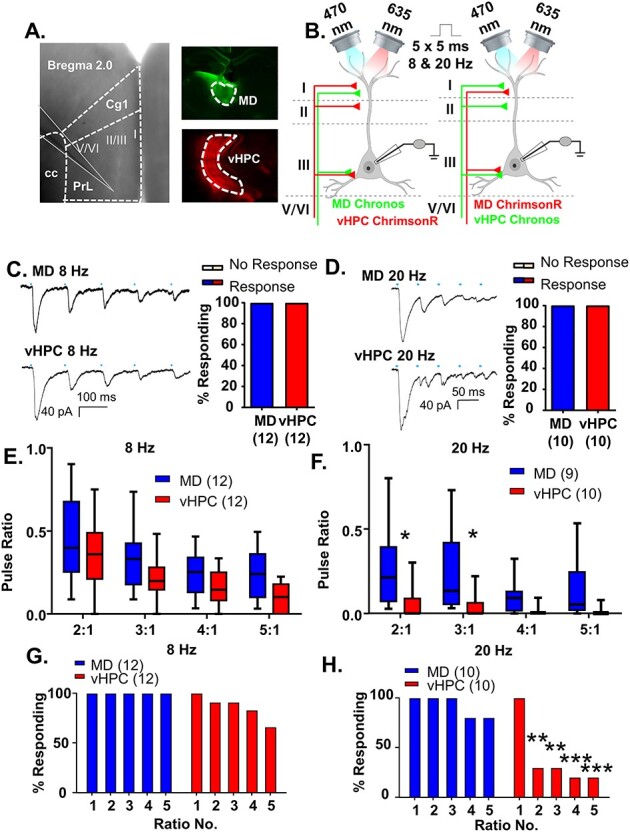
MD and vHPC inputs converge onto layer 2/3 mPFC pyramidal neurons but display different frequency-dependent synaptic dynamics. (*A*) Low power representative image indicating recording location in layer 2/3 of prelimbic mPFC (left; Prl is prelimbic cortex, Cg1 is cingulate cortex and cc is corpus callosum). Low power images illustrating injection spread of Chronos within the mediodorsal thalamus (MD, top right, green) and ChrimsonR within the ventral hippocampus (vHPC, bottom right, red). (*B*) Cartoon schematic illustrating the experiment. Animals expressing either Chronos or ChrimsonR in the MD and the opposite opsin in the vHPC were stimulated with five, 5-ms pulses of 470 or 635 nm light, respectively, at a frequency of 8 or 20 Hz while recording from mPFC pyramidal neurons. (*C*) Example traces of currents evoked in layer 2/3 mPFC pyramidal cells in response to five, 5-ms pulses of 470 or 635 nm light stimulating MD and vHPC terminals at 8 Hz. (*D*) Example traces of currents evoked in layer 2/3 mPFC pyramidal cells in response to five, 5-ms pulses of 470 or 635 nm light stimulating MD and vHPC terminals at 20 Hz. (*E*) The ratio of responses to repeated light stimuli relative to the first stimulus was similar for stimulation of MD or vHPC terminals at 8 Hz. There was no significant difference between the ratio of any of the responses for stimulation of the MD versus vHPC in the Bonferroni-corrected posthoc analysis. (*F*) The ratio of responses to repeated light stimuli relative to the first stimulus differed for stimulation of MD versus vHPC terminals at 20 Hz. There was a significant difference between the ratio of the second and third stimulation relative to the first for the MD versus vHPC in the Bonferroni-corrected posthoc analysis. (*G*) The percent of cells showing a significant (>2× standard deviation of the baseline noise) response to each pulse of light for vHPC versus MD was slightly attenuated for pulses 2–5 for stimulation of the vHPC relative to the MD at 8 Hz. (*H*) The percent of cells showing a significant response to each pulse of light for vHPC versus MD at 20 Hz. A large number of cells failed to show a significant response to repeated stimulations of vHPC terminals; this was not the case for stimulation of the MD. Differences in the percent of cells responding to stimulation of the 2 inputs based on Chi-square analysis are indicated by significance stars. ^*^P < 0.05; ^**^P < 0.01; ^***^P < 0.001.

### Experimental Design and Statistical Analysis

For single injection experiments to compare cross-reactivity of ChrimsonR and Chronos to blue (470 nm) and red (635 nm) light, we used 3–6 cells per condition from 1 to 2 mice (all male). For characterization of ChrimsonR and Chronos kinetics we used between 10 and 15 cells per condition from between 2 and 5 mice (all male). To compare MD and vHPC inputs to pyramidal cells, we used 10–12 cells per condition from 6 mice (5 males, 1 female). To compare MD and vHPC inputs to PV cells, we used 8–10 cells per condition from 5 mice (all male). To compare MD and vHPC inputs to VIP cells, we used 6 cells per condition from 2 mice (1 male, 1 female). For single injection experiments of ChR2(H134R) (including those at both 25° and 32 °C) in the MD or vHPC, we used 20 cells for the MD from 6 mice (5 male, 1 female) and 16 cells for the vHPC from 6 mice (all male).

For analysis of effects of ChR2 variant stimulated and stimulation number, we used a two-way repeated measure ANOVA for continuous variables (le-PSC amplitude and ratios). For these analyses, we looked at both main effects of the two independent variables as well as if there was an interaction. Statistical significance was denoted in the figures based on Bonferroni-corrected posthoc analysis to assess the effect of ChR2 variant at each repeated stimulus. For analysis of effects of brain region stimulated, we used a Chi-square comparison for categorical variables (responding/non-responding). For analysis of effects of brain region stimulated and stimulation number, we used a two-way repeated measure ANOVA for continuous variables (le-PSC amplitude and ratios). For these analyses, we looked at both main effects of the two independent variables as well as if there was an interaction. Statistical significance was denoted in the figures based on Bonferroni-corrected posthoc analysis to assess the effect of brain region at each repeated stimulus. Statistical analysis was conducted using GraphPad Prism 8.

## Results

### Establishing Dual Stimulation Parameters for Activation of Chronos and ChrimsonR

To address directly whether MD and vHPC inputs converge onto the same populations of mPFC pyramidal cells, we implemented a dual optogenetic stimulation strategy using the ChrimsonR and Chronos system. This dual stimulation strategy relies on the separation of wavelength activation of the two opsins, with ChrimsonR being red-shifted (peak activation with 635 nm) while Chronos is blue-shifted (peak activation with 470 nm) (Klapoetke et al. 2014). To validate this dual stimulation strategy in our preparation, we made whole cell voltage clamp recordings at −70 mV from mPFC pyramidal cells in mice where either Chronos or ChrimsonR was expressed in the MD while stimulating with 470 or 635 nm light while progressively increasing light intensity (Fig. 1). As previously reported, we found that both blue and red shifted ChR2 variants are activated by 470 nm light, though with different efficiency (Klapoetke et al. 2014). We therefore optimized the stimulation conditions to avoid cross-activation. 470 nm light delivered at 1% of the maximum LED intensity was sufficient to activate Chronos, while avoiding cross-activation of ChrimsonR as 3/3 Chronos-expressing terminals stimulated with 470 nm light at 1% LED intensity produced a significant le-PSC versus 0/6 ChrimsonR-expressing terminals (Fig. 1*B*). In contrast, we found that 635 nm light only activated ChrimsonR as 0/3 Chronos-expressing terminals stimulated with 635 nm light at 100% LED intensity produced a significant le-PSC (Fig. 1*D*).

### Chronos and ChrimsonR Show Comparable Response Properties

In order to utilize this dual opsin system to interrogate the frequency-dependent synaptic dynamics of MD and vHPC inputs, we needed to establish that both opsins produced similar frequency-dependent responses. To this end, we measured mPFC pyramidal neuron responses to five repeated stimulations at 8 and 20 Hz of MD terminals expressing either ChrimsonR or Chronos, respectively (Fig. 2*A*). 8 Hz was selected because it falls within the theta-frequency range (4–12 Hz), which is commonly associated with endogenous vHPC firing patterns entrained with prefrontal cells in studies utilizing in vivo neural recordings (Jones and Wilson 2005; Siapas et al. 2005; Sigurdsson et al. 2010; Adhikari et al. 2011; Padilla-Coreano et al. 2016; Padilla-Coreano et al. 2019). 20 Hz was originally chosen because it is a common frequency for assessing paired pulse ratios (Graziane and Dong 2016); additionally, beta-range (20 Hz) frequencies have been shown to be relevant for MD–PFC communication during working memory maintenance (Parnaudeau et al. 2013).

Using stimulation intensities of 1% for 470 nm and between 2 and 90% for 635 nm, the amplitudes of the le-PSCs following stimulation of either Chronos or ChrimsonR-expressing MD terminals did not differ by ChR2 variant at either 8 Hz (Fig. 2*B*,*D* and Supplementary Fig. 1*A*; Chronos *n* = 15 cells; ChrimsonR *n* = 10 cells; two-way rmANOVA, no main effect of ChR2 variant *F*(1, 23) = 0.2256, *P* = 0.6393) or 20 Hz (Fig. 2*C*,*E* and Supplementary Fig. 1*B*; Chronos *n* = 10 cells; ChrimsonR *n* = 10 cells; two-way rmANOVA, no main effect of ChR2 variant *F*(1, 18) = 1.366, *P* = 0.2578). Similar to previously published characterizations of the kinetics of Chronos and ChrimsonR (Klapoetke et al. 2014), we found that their degree of desensitization in response to repeated stimulations at 8 Hz was similar (Fig. 2*F* and Supplementary Fig. 1*C*; Chronos *n* = 15 cells; ChrimsonR *n* = 10 cells; 2-way rmANOVA, no main effect of ChR2 variant *F*(1, 23) = 0.05880, *P* = 0.8106; main effect of ratio number *F*(3, 69) = 11.14, *P* < 0.0001; ChR2 variant by ratio number interaction *F*(3, 69) = 3.706, *P* = 0.0156 but no significant Bonferroni-corrected posthoc differences between the ChR2 variants at any pulse). In response to stimulation at 20 Hz, ChrimsonR was slightly but significantly more desensitizing (Fig. 2*G* and Supplementary Fig. 1*D*; Chronos *n* = 10 cells; ChrimsonR *n* = 10 cells; 2-way rmANOVA, main effect of ChR2 variant *F*(1, 18) = 4.481, *P* = 0.0485; main effect of ratio number *F*(3, 54) = 5.981, *P* = 0.0013; no ChR2 variant by ratio number interaction *F*(3, 54) = 2.592, *P* = 0.0621. However, there was a significant Bonferroni-corrected posthoc difference between ChR2 variants at the first pulse ratio tested (ratio 2:1, multiplicity-adjusted ^**^P = 0.0046). To offset this slight difference in the frequency-dependent synaptic dynamics, we varied whether Chronos or ChrimsonR was expressed in the MD or vHPC, respectively, as noted below.

**Figure 4:**
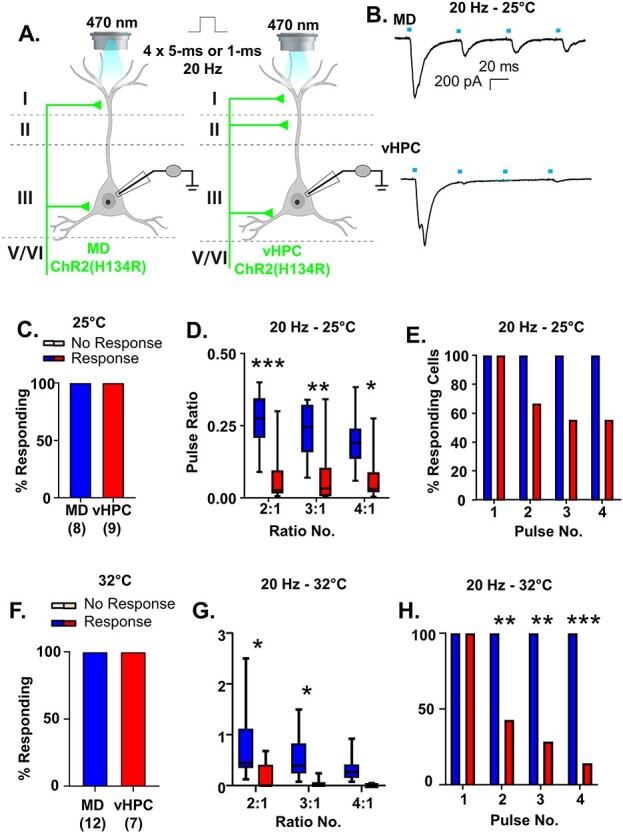
vHPC inputs are more desensitizing than MD inputs regardless of recording temperature and stimulation duration. (*A*) Cartoon schematic illustrating the experiment. Slices containing the PFC from animals expressing ChR (H134R) in either the MD or vHPC were stimulated with four pulses of 470 nm light at 20 Hz. Pulses were either 5 ms (*B*–*E*) or 1 ms (*F*–*H*). (*B*) Example traces of currents evoked in layer 2/3 mPFC pyramidal neurons in response to four 5-ms pulses of 470 nm light stimulating either the MD or the vHPC at 20 Hz while recording at 25 °C. (*C*) All cells tested showed a light-evoked post synaptic response to stimulation of either MD or vHPC inputs with the first pulse of light. (*D*) The ratio of responses to repeated light stimuli relative to the first stimulus differed for stimulation of MD versus vHPC terminals at 20 Hz. There was a significant difference between the ratio of the second and third and fourth stimulation relative to the first for the MD versus vHPC in the Bonferroni-corrected posthoc analysis. (*E*) The percent of cells showing a significant (>2× standard deviation of the baseline noise) response to each pulse of light for vHPC versus MD was attenuated (though not significantly) for pulses 2–4 for stimulation of the vHPC relative to the MD at 20 Hz. (*F*) When the recordings took place at 32 °C with a shorter light stimulation duration (1 ms), similar results were obtained. All pyramidal cells tested showed a light-evoked post synaptic response to stimulation of either MD or vHPC inputs with the first pulse of light. (*G*) Under these conditions, the ratio of responses to repeated light stimuli relative to the first stimulus differed for stimulation of MD versus vHPC terminals at 20 Hz. There was a significant difference between the ratio of the second and third and fourth stimulation relative to the first for the MD versus vHPC in the Bonferroni-corrected posthoc analysis. (*H*) The percent of cells showing a significant (>2× standard deviation of the baseline noise) response to each pulse of light for vHPC versus MD was significantly attenuated for pulses 2–4 for stimulation of the vHPC relative to the MD at 20 Hz. ^*^P < 0.05, ^**^P < 0.01, ^***^P < 0.001.

### MD and vHPC Inputs Converge onto Layer 2/3 mPFC Pyramidal Neurons but Display Different Frequency-Dependent Synaptic Dynamics

Having established our parameters for this dual stimulation approach, we directly compared inputs from the MD and vHPC to mPFC projection neurons. To counterbalance opsin use, we injected the MD with Chronos and the vHPC with ChrimsonR in one set of animals, while in another set of animals we injected the vHPC with Chronos and the MD with ChrimsonR. We recorded mPFC pyramidal cells from layer 2/3 while stimulating MD or vHPC inputs (Fig. 3*A*,*B* and Supplementary Fig. 2).

All recorded pyramidal cells received input from both the MD and vHPC. All 12 MD and vHPC cells stimulated at 8 Hz (Fig. 3*C*) and all 10 MD and vHPC cells stimulated at 20 Hz (Fig. 3*D*) showed a significant le-PSC in response to the first pulse of light. At either 8 or 20 Hz, the response to repeated stimulations of either input was consistently desensitizing, similar to what has been seen for long-range excitatory inputs to PV cells in V1 (Lu et al. 2014). We next compared the synaptic dynamics of MD and vHPC inputs at both frequencies. We found that at 8 Hz, vHPC inputs were slightly more desensitizing than MD ones as seen in the ratio of le-PSC responses to subsequent pulses of light relative to the first pulse (Fig. 3*E* and Supplementary Fig. 3*A*; MD and vHPC *n* = 12 cells; two-way rmANOVA, no main effect of region *F*(1, 22) = 3.399, *P* = 0.0788; main effect of ratio number *F*(3, 66) = 26.07, *P* < 0.0001; no region by ratio number interaction, *F*(3, 66) = 0.5175, *P* = 0.6717 nor any significant Bonferroni-corrected posthoc differences between the brain regions at each ratio). At 20 Hz, differences in desensitization between brain regions were much more pronounced, as seen in a comparison of the ratio of le-PSC responses to subsequent pulses of light relative to the first pulse (Fig. 3*F* and Supplementary Fig. 3*B*; MD *n* = 9 cells and vHPC *n* = 10 cells; two-way ANOVA, main effect of region *F*(1, 17) = 6.309, *P* = 0.0224; main effect of ratio number *F*(3, 51) = 8.679, *P* < 0.0001; a region by ratio number interaction *F*(3, 51) = 3.051, *P* = 0.0367). Bonferroni-corrected posthoc comparisons of brain regions at each ratio revealed the vHPC responses were significantly more desensitized for ratios 2:1 and 3:1 (Bonferroni posthoc MD vs. vHPC: ratio 2:1, multiplicity-adjusted ^*^P = 0.0108, ratio 3:1, multiplicity-adjusted ^*^P = 0.0238, ratio 4:1, multiplicity-adjusted *P* = 0.9015, ratio 5:1, multiplicity-adjusted *P* = 0.2562). This desensitization of vHPC inputs to stimulation at 20 Hz was so pronounced that while MD inputs consistently provoked significant le-PSCs in response to repeated stimulations, vHPC inputs desensitized to the point that only 20% of the pyramidal cells recorded showed a significant le-PSC after the first stimulation (Fig. 3*H*; Chi-square comparison of percent responding MD vs. vHPC: pulse 1, *P* > 0.9999; pulse 2, ^**^P = 0.0031; pulse 3, ^**^P = 0.0031; ^***^pulse 4, *P* = 0.0007; ^***^pulse 5, *P* = 0.0007). By comparison, all MD and the majority of vHPC inputs provoked significant le-PSCs in response to repeated stimulations at 8 Hz (Fig. 3*G*; Chi-square comparison of percent responding MD vs. vHPC: pulse 1, *P* > 0.9999; pulse 2, *P* > 0.9999; pulse 3, *P* > 0.9999; pulse 4, *P* = 0.48; pulse 5, *P* = 0.09). To affirm that the opsin used to stimulate each of pathways did not influence these results, we compared the pulse ratios evoked by stimulation of the MD or vHPC at 8 or 20 Hz by opsin type and found they did not differ (data not shown).

As further confirmation of our dual stimulation results, when we expressed the commonly used H134R variant of ChR2 individually in the MD or vHPC and stimulated these individual inputs using stimulation conditions comparable to the dual stimulation ones (5-ms stimulation pulses recorded at 25 °C), we found that all layer 2/3 pyramidal cells tested displayed significant responses to stimulation of either input (Fig. 4*C*; *n* = 8/8 cells for MD, 9/9 cells for vHPC). Furthermore, we found that vHPC inputs displayed greater frequency-dependent synaptic depression at 20 Hz than MD inputs (Fig. 4*D*,*E*). While repeated stimulation of both MD and vHPC inputs at 20 Hz resulted in postsynaptic responses that decreased in amplitude, this depression was significantly greater for le-PSCs generated in response to stimulation of the vHPC (Fig. 4*D* and Supplementary Fig. 4*A*; MD *n* = 8 cells; vHPC *n* = 9 cells; 2-way rmANOVA, main effect of region *F*(1, 15) = 13.58, *P* = 0.0022; main effect of ratio number *F*(2, 30) = 5.646, *P* = 0.0083 and a region by ratio number interaction *F*(2, 30) = 4.682, *P* = 0.0170). Bonferonni-corrected posthoc comparisons between the regions for each of the response ratios revealed significant differences between stimulation of the MD and vHPC terminals for all ratios examined (Bonferroni posthoc MD vs. vHPC, ratio 2:1, multiplicity-adjusted ^***^P = 0.0003, ratio 3:1, multiplicity-adjusted ^**^P = 0.0035, and ratio 4:1, multiplicity-adjusted ^*^P = 0.0199). The desensitization of vHPC inputs to repeated 20 Hz stimulation was such that up to 50% of responses were not statistically distinguishable from the baseline noise, whereas stimulation of the MD inputs consistently evoked detectable postsynaptic le-PSCs (Fig. 4*E*; Chi-square comparison of percent responding MD vs. vHPC: pulse 1, *P* > 0.9999; pulse 2, *P* = 0.2059; pulse 3, *P* = 0.0824; pulse 4, *P* = 0.0824). This difference in synaptic dynamics of the two inputs at 20 Hz was not an artifact of the stimulation conditions as similar results were obtained recording with a shorter pulse duration (1 ms) at 32 °C (Fig. 4*F*–*H*). Specifically, 12 of 12 pyramidal neurons responded to stimulation of MD inputs and 7 of 7 pyramidal cells responded to stimulation of vHPC inputs (Fig. 4*F*). As in the recordings made at 25 °C, repeated stimulation of both MD and vHPC inputs at 20 Hz was desensitizing. In line with previous observations, the degree of desensization was significantly greater for vHPC inputs than MD inputs (Fig. 4*G* and Supplementary Fig. 4*B*; MD *n* = 12 cells; vHPC *n* = 7 cells; two-way rmANOVA, main effect of region *F*(1, 17) = 7.439, *P* = 0.0143; main effect of ratio number *F*(2, 34) = 7.682, *P* = 0.0018 and no region by ratio number interaction *F*(2, 34) = 1.484, *P* = 0.2409). Bonferonni-corrected posthoc comparisons of the response ratios by region revealed significant differences between stimulation of the MD and vHPC terminals for ratios 2:1 and 3:1 (Bonferroni posthoc MD vs. vHPC, ratio 2:1, multiplicity-adjusted ^*^P = 0.0104, ratio 3:1, multiplicity-adjusted ^*^P = 0.0451, and ratio 4:1, multiplicity-adjusted *P* = 0.2654). The desensitization of vHPC inputs to repeated 20 Hz stimulation was such that up to 85% of responses were not statistically distinguishable from the baseline noise, whereas stimulation of the MD inputs consistently evoked detectable postsynaptic le-PSCs (Fig. 4*H*; Chi-square comparison of percent responding MD vs. vHPC: pulse 1, *P* > 0.9999; pulse 2, ^**^P = 0.0090; pulse 3, ^**^P = 0.0018; pulse 4, ^***^P = 0.0003). These data confirmed our findings from the dual stimulation experiments; while MD and vHPC inputs innervate all recorded layer 2/3 pyramidal cells in the ipsilateral prelimbic cortex, in response to stimulation at 20 Hz, the frequency-dependent synaptic dynamics of both inputs differ.

Altogether, these data support a model in which the layer 2/3 mPFC cells receive both MD and vHPC innervation but respond differently to input from each projection.

### MD Inputs Preferentially Excite mPFC PV-Interneurons Relative to vHPC Inputs

**Figure 5:**
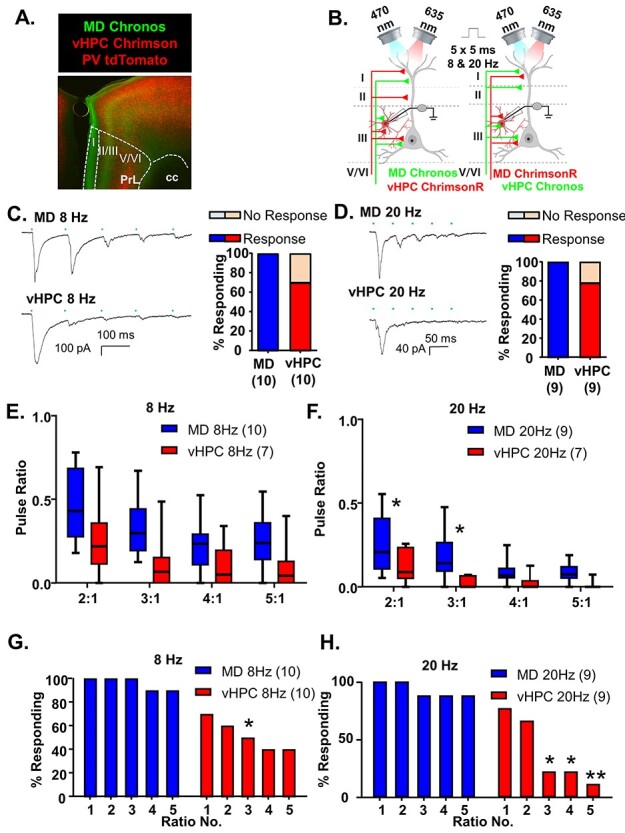
MD inputs preferentially excite mPFC PV-interneurons relative to vHPC inputs. (*A*) Representative 5× photomicrograph illustrating MD inputs labeled with Chronos-EGFP (green), PV interneurons fluorescently labeled with tdTomato (red) and vHPC inputs labeled with ChrimsonR-tdTomato (red). PrL is prelimbic and cc is corpus callosum. (*B*) Cartoon schematic illustrating the experiment. Animals expressing either Chronos or ChrimsonR in the MD and the opposite opsin in the vHPC were stimulated with five, 5-ms pulses of 470 or 635 nm light, respectively, at a frequency of 8 or 20 Hz while recording from mPFC PV interneurons. (*C*) Example traces of currents evoked in layer 2/3 mPFC PV interneurons in response to five, 5-ms pulses of 470 or 635 nm light stimulating MD and vHPC terminals at 8 Hz. All cells tested showed a light-evoked post synaptic response to stimulation of MD terminals with the first pulse of light, while only 70% showed a significant response to stimulation of vHPC terminals with the first light pulse. (*D*) Example traces of currents evoked in layer 2/3 mPFC pyramidal cells in response to five, 5-ms pulses of 470 or 635 nm light stimulating MD and vHPC terminals at 20 Hz. All cells tested showed a light-evoked post synaptic response to stimulation of MD terminals with the first pulse of light, while only 78% showed a significant response to stimulation of vHPC terminals with the first light pulse. (*E*) The ratio of responses to repeated light stimuli relative to the first stimulus was similar for stimulation of MD or vHPC terminals at 8 Hz. There was no significant difference between the response ratio of any of the responses for stimulation of the MD versus vHPC at 8 Hz in the Bonferroni-corrected posthoc analysis. (*F*) The ratio of responses to repeated light stimuli relative to the first stimulus differed for stimulation of MD versus vHPC terminals at 20 Hz. There was a significant difference between the ratio of the second and third stimulation relative to the first for the MD versus vHPC in the Bonferroni-corrected posthoc analysis. (*G*) The percent of cells showing a significant (>2× standard deviation of the baseline noise) response to each pulse of light was decreased following all pulses of stimulation of the vHPC relative to the MD at 8 Hz and at 20 Hz (*H*). Differences in the percent of cells responding to stimulation of the 2 inputs based on Chi-square analysis are indicated by significance stars. ^*^P < 0.05; ^**^P < 0.01.

**Figure 6:**
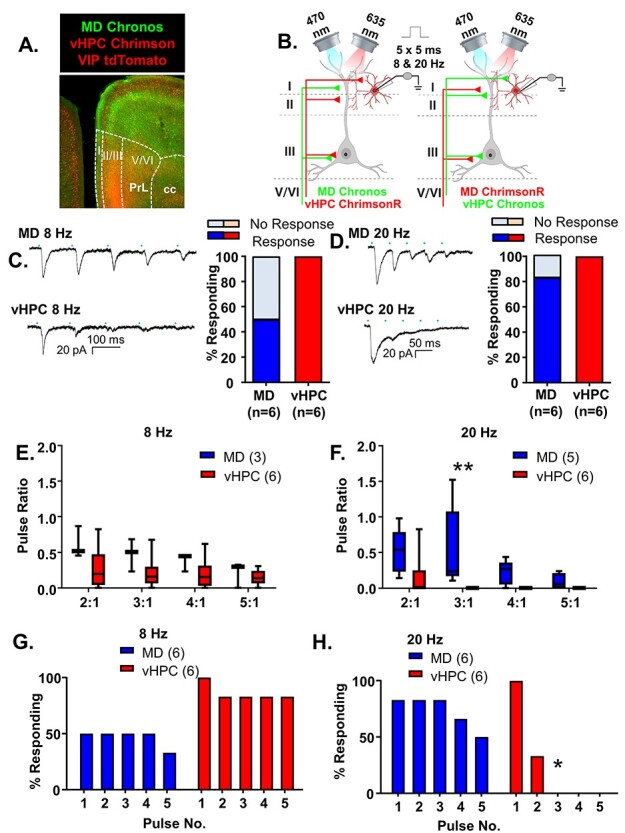
vHPC inputs preferentially excite mPFC VIP-interneurons relative to MD inputs. (*A*) Representative 5× photomicrograph illustrating MD inputs labeled with Chronos-EGFP (green), VIP interneurons fluorescently labeled with tdTomato (red) and vHPC inputs labeled with ChrimsonR-tdTomato (red). PrL is prelimbic and cc is corpus callosum. (*B*) Cartoon schematic illustrating the experiment. Animals expressing either Chronos or ChrimsonR in the MD and the opposite opsin in the vHPC were stimulated with five, 5-ms pulses of 470 or 635 nm light, respectively, at a frequency of 8 or 20 Hz while recording from mPFC VIP interneurons. (*C*) Example traces of currents evoked in layer 2/3 mPFC VIP interneurons in response to five, 5-ms pulses of 470 or 635 nm light stimulating MD and vHPC terminals at 8 Hz. All cells tested showed a light-evoked post synaptic response to stimulation of vHPC terminals with the first pulse of light, while only 50% showed a significant response to stimulation of MD terminals with the first light pulse. (*D*) Example traces of currents evoked in layer 2/3 mPFC pyramidal cells in response to five, 5-ms pulses of 470 or 635 nm light stimulating MD and vHPC terminals at 20 Hz. All cells tested showed a light-evoked postsynaptic response to stimulation of vHPC terminals with the first pulse of light, while only 83% showed a significant response to stimulation of MD terminals with the first light pulse. (*E*) The ratio of responses to repeated light stimuli relative to the first stimulus did not differ for stimulation of MD versus vHPC terminals at 8 Hz. There was no significant difference between the ratio of any of the responses for stimulation of the MD versus vHPC at 8 Hz in the Bonferroni-corrected posthoc analysis. (*F*) The ratio of responses to repeated light stimuli relative to the first stimulus differed for stimulation of MD versus vHPC terminals at 20 Hz. There was a significant difference between the ratio of third pulse relative to the first for stimulation of the MD versus vHPC at 20 Hz in the Bonferroni-corrected posthoc analysis. (*G*) The percent of cells showing a significant (>2× standard deviation of the baseline noise) response to each pulse of light was decreased following all pulses of stimulation of the MD relative to the vHPC at 8 Hz and at 20 Hz (*H*). Differences in the percent of cells responding to stimulation of the two inputs based on Chi-square analysis are indicated by significance stars. ^*^P < 0.05; ^**^P < 0.01.

We next asked whether MD and vHPC inputs would show similar convergence and synaptic dynamics onto PFC interneurons. In order to record from different genetically identified populations of interneurons, we made injections of ChrimsonR and Chronos into the MD and vHPC in mice where a fluorescent reporter (tdTomato) was expressed in either the PV or the VIP population of interneurons by crossing Ai9 mice (tdTomato reporter mice) to either PV-Cre (Fig. 5*A*,*B*) or the VIP-Cre mice (Fig. 6*A*,*B*). We also confirmed the identity of these cells as significantly different than that of pyramidal cells based on their smaller capacitance measurements, indicative of their smaller size (Supplementary Fig. 5; One-way ANOVA, *F*(2, 23) = 21.06, *P* < 0.0001. Bonferroni posthoc PV vs. Pyr, multiplicity-adjusted ^****^P < 0.0001; VIP vs. Pyr, multiplicity-adjusted ^***^P = 0.0001). Importantly, to facilitate comparison of results from these different cell populations, the recordings from pyramidal neurons (Fig. 3) were made in these same slices, enabling us to control for possible differences in the injection and/or infection efficiency.

Stimulation of MD inputs consistently evoked postsynaptic responses in PV interneurons (Fig. 5*C*,*D*). However, the initial stimulation of vHPC inputs only resulted in le-PSCs in 7 of 10 PV interneurons (70%) in 8 Hz stimulation trials and in 7 of 9 PV interneurons (78%) in 20 Hz stimulation trials (Fig. 5*C*,*D*). Responses were recorded from neighboring pyramidal cells in all slices where PV cells failed to show a response to vHPC stimulation, indicating this lack of response is not due to differences in injection efficiency or targeting. Moreover, there was no significant correlation between the size of the pyramidal cell response and the size of the interneuron response following stimulation of either MD or vHPC inputs, which would be expected if the density of viral expression was determining the size of the le-PSCs (Supplementary Fig. 6; linear regression *F*(1, 28) = 2.55, *P* = 0.12). In response to repeated stimulations at 8 Hz, both MD and vHPC inputs desensitized. When we compared the ratio of responses with repeated 8 Hz stimulations relative to the first to measure the degree of desensitization for MD versus vHPC inputs, we observed that vHPC inputs were more desensitized than MD inputs (Fig. 5*E* and Supplementary Fig. 7*A*; MD *n* = 10 cells and vHPC *n* = 7 cells; two-way rmANOVA, main effect of region *F*(1, 15) = 5.358, *P* = 0.0352; main effect of ratio number *F*(3, 45) = 14.99, *P* < 0.0001; no interaction of region by ratio number *F*(3, 45) = 0.529, *P* = 0.6646 nor any Bonferroni-corrected posthoc differences between the regions for any of the ratios measured). Both inputs also desensitized following 20 Hz stimulation and this desensitization was significantly greater for vHPC inputs (Fig. 5*F* and Supplementary Fig. 7*B*; MD *n* = 8 cells and vHPC *n* = 7 cells; 2-way rmANOVA, main effect of region *F*(1, 13) = 7.263, *P* = 0.0184; main effect of ratio number *F*(3, 39) = 11.68, *P* < 0.0001; no interaction of region by ratio number *F*(3, 39) = 1.657, *P* = 0.1920). Bonferroni-corrected posthoc comparisons revealed significant differences between stimulation of the MD and vHPC terminals for ratios 2:1 and 3:1 (ratio 2:1, multiplicity-adjusted ^*^P = 0.0432; ratio 3:1, multiplicity-adjusted ^*^P = 0.0110). We then compared the percentage of PV neurons that showed a significant le-PSC in response to each stimulation of either MD or vHPC input. The fraction of neurons responding to repeated pulses was attenuated for vHPC stimulations relative to MD ones at 8 Hz (Fig. 5*G*; Chi-square comparison of percent responding MD vs. vHPC: pulse 1, *P* = 0.2105; pulse 2, *P* = 0.0867; pulse 3, ^*^P = 0.0325; pulse 4, *P* = 0.0573; pulse 5, *P* = 0.0573). This attenuation was more significant at 20 Hz (Fig. 5H, Chi-square comparison of percent responding MD vs. vHPC: pulse 1, *P* = 0.4706; pulse 2, *P* = 0.2509; pulse 3, ^*^P = 0.0152; pulse 4, ^*^P = 0.0152; pulse 5, ^**^P = 0.0034).

### vHPC Inputs Preferentially Excite mPFC VIP-Interneurons Relative to MD Inputs

Next, we recorded VIP interneurons while stimulating MD or vHPC inputs with the same approach described above (Fig. 6*A*,*B*). In contrast to PV interneurons, which appear preferentially innervated by MD inputs, VIP interneurons were preferentially innervated by vHPC inputs. Stimulation of vHPC inputs reliably generated responses in all VIP interneurons sampled; however, the initial stimulation of MD inputs only resulted in le-PSCs in 50% of VIP interneurons in 8 Hz stimulation trials and in 80% of VIP interneurons in 20 Hz stimulation trials (Fig. 6*C*,*D*). When we compared the response of MD versus vHPC inputs with repeated stimulations at 8 Hz, we found that both inputs desensitized but were not significantly different (Fig. 6*E* and Supplementary Fig. 8*A*; MD *n* = 3 cells and vHPC *n* = 6 cells; 2-way rmANOVA, no main effect of region *F*(1, 7) = 2.198, *P* = 0.1817; main effect of ratio number *F*(3, 21) = 11.57, *P* = 0.0001; interaction of region by ratio number *F*(3, 21) = 3.411, *P* = 0.0363). However, there were no significant differences in the Bonferroni-corrected posthoc comparisons between regions at any ratio number. Both inputs also desensitized following 20 Hz stimulation, and the degree of desensitization was significantly greater for vHPC than MD inputs, similar to what was seen with pyramidal and PV cells (Fig. 6*F* and Supplementary Fig. 8*B*; MD *n* = 5 cells and vHPC *n* = 6 cells; 2-way rmANOVA, main effect of region *F*(1, 9) = 12.29, *P* = 0.0067; main effect of ratio number *F*(3, 27) = 3.039, *P* = 0.0461; no region by ratio number interaction *F*(3, 27) = 1.596, *P* = 0.2134). There was a significant difference in the Bonferroni-corrected posthoc comparison between regions at ratio 3:1 (ratio 3:1, multiplicity-adjusted ^**^P = 0.0063). There was no significant difference in the percent of VIP cells responding to repeated stimulation of the MD versus vHPC inputs at 8 Hz (Fig. 5*G*, Chi-square comparison percent responding MD vs. vHPC: pulse 1, *P* = 0.1818; pulse 2, *P* = 0.5455; pulse 3, *P* = 0.5455; pulse 4, *P* = 0.5455; pulse 5, *P* = 0.2424). Although VIP cells were initially more likely to receive input from the vHPC, at 20 Hz, the degree of desensitization of vHPC inputs was such that stimulation of this input was significantly less likely to provoke a response to repeated stimulations than MD inputs (Fig. 6H, Chi-square comparison percent responding MD versus vHPC: pulse 1, *P* > 0.9999; pulse 2, *P* = 0.2424; pulse 3, ^*^P = 0.0152; pulse 4, *P* = 0.0606; and pulse 5, *P* = 0.1818). As previously described, we successfully recorded responses in pyramidal cells following stimulation of MD inputs in the same slices from VIP interneurons that failed to respond to MD stimulation, and the amplitude of the interneuron and pyramidal cell responses was not significantly correlated (Supplementary Fig. 6), indicating that the differential response of PV and VIP cells to MD versus vHPC input did not reflect differences in infection or injection efficiency in the various preparations. As further confirmation of this, we compared the le-PSC amplitude evoked in these neighboring pyramidal cells in slices from PV- and VIP-Cre mice and found the response amplitudes in pyramidal cells were comparable for stimulation of MD input (PV-Cre *n* = 7 cells, mean response ± SD = 465.6 ± 304.1 pA, and VIP-Cre *n* = 3 cells, mean response ± SD = 835.4 ± 607.7 pA; unpaired *t*-test *P* = 0.1797) or vHPC input (PV-Cre *n* = 7 cells, mean response ± SD = 433.6 ± 360 pA, and VIP-Cre *n* = 3 cells, mean response ± SD = 239.1 ± 149.9 pA; unpaired *t*-test *P* = 0.3957). Although the size of these groups is small, the mean pyramidal cell responses to stimulation of MD inputs were larger in VIP-Cre mice, where VIP interneurons appeared to receive less MD input.

### Onset Latency Analysis Indicates all Measured Responses Contain a Monosynaptic Component

**Figure 7:**
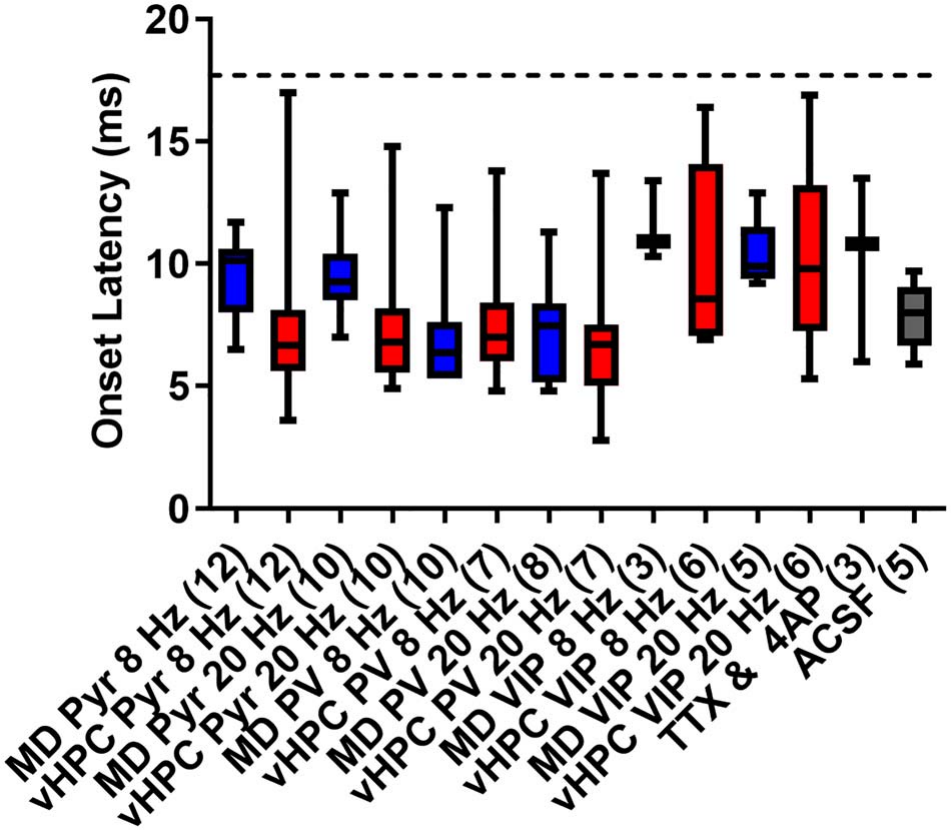
Analysis of the onset latency for light-evoked postsynaptic currents indicates all measured responses contain a monosynaptic component. Box and whisker plots showing the median, as well as the minimum and maximum values of the response onset latency following stimulation of MD or vHPC inputs at 8 or 20 Hz for all cells analyzed in this paper. Note, the onset latencies for all responses of cells included in this analysis fall below the threshold for monosynaptic transmission defined as less than two standard deviations from the mean of the TTX + 4-AP condition (17.7 ms, dotted line).

Finally, to determine whether the excitatory le-PSC responses we were measuring contained a monosynaptic component and were mediated by direct inputs from either the MD or the vHPC to mPFC cells, we measured the onset latency of the responses. To this end, we first assessed the onset latency of le-PSC currents evoked under conditions that isolate monosynaptic transmission by applying tetrodotoxin (TTX) in combination with 4-aminopuridine (4-AP) to the bath. Tetrodotoxin blocks action potentials so that the only source of neurotransmitter release is via direct activation of ChR2 in the terminals. 4-AP was added to the bath to increase the amplitude of le-PSCs evoked under these conditions as le-PSC amplitude is greatly reduced in the presence of TTX. These conditions were not used for all experiments as they also decreased the amplitude of the evoked, postsynaptic responses, biasing towards not detecting a significant connection when one might in fact be there. Monosynaptic le-PSCs evoked under conditions of TTX and 4-AP had a mean onset latency of 10.10 ms with a standard deviation of 3.799 ms. We used the mean of the monosynaptic latencies plus two times the standard deviation of these latencies (17.7 ms) as the threshold below which we considered onset latencies under non-TTX experimental conditions as likely monosynaptic (Fig. 7). Based on this criteria, we found that all of our evoked le-PSCs likely contained a monosynaptic component (Fig. 7).

## Discussion

### MD and vHPC Inputs Converge onto Layer 2/3 mPFC Pyramidal Cells

One primary finding is that MD and vHPC inputs converge onto all layer 2/3 pyramidal cells in the prelimbic mPFC. While this result is consistent with previous work demonstrating widespread functional connectivity from the MD and vHPC onto layer 2/3 pyramidal cells of the prelimbic mPFC (Parent et al. 2010; Little and Carter 2012), our study is the first to directly test the functional convergence of MD and vHPC inputs onto “individual” mPFC local circuit elements using the dual input circuit-mapping strategy of ChrimsonR and Chronos. The widespread functional convergence between the two inputs onto L2/3 PFC neurons is striking, given that optogenetic inhibition studies indicate clear distinctions in the neural correlates and behavioral relevance of the two connections (Adhikari et al. 2011; Spellman et al. 2015; Padilla-Coreano et al. 2016; Bolkan et al. 2017; Schmitt et al. 2017). This result suggests that simple anatomical connectivity, at least onto layer 2/3 pyramidal cells, does not underlie these differential behavioral requirements.

It may be that the relative strength of these inputs onto individual pyramidal cells is an important determinant in their behavioral distinction. It is difficult to compare directly the amplitudes of light evoked currents from MD versus vHPC inputs to layer 2/3 neurons since this is determined not only by the synaptic strengths but also by the amount of virus injected and the intensity of light stimulation. A recent paper challenged the importance of vHPC input to layer 2/3 in prelimbic mPFC by suggesting the connections are weak onto layer 2/3 cells in prelimbic cortex relative to those onto pyramidal cells in layer 5 (Liu and Carter 2018). In addition, a separate study found that MD inputs to pyramidal cells in mPFC appeared to be stronger onto layer 2/3 versus layer 5 (Collins et al. 2018). While we did not directly compare the relative strength of inputs from either MD or vHPC to layer 2/3 versus 5 in our study, our observation of consistent connectivity in both singly injected animals expressing ChR2 exclusively in the MD or vHPC, as well as in dually injected mice, argues that both regions provide an important source of information to layer 2/3 cells in this region. Nevertheless, a bias of input strength from the MD onto layer 2/3 versus 5 and from the vHPC onto layer 5 versus 2/3 might be a mechanism allowing some anatomical segregation in this circuitry.

### MD and vHPC Inputs Show Bias in their Targeting of Layer 2/3 mPFC PV and VIP Interneurons

In contrast to connectivity to pyramidal neurons, we found a bias in connections onto distinct populations of local interneurons. In particular, the populations expressing PV and VIP are of interest given that they are nonoverlapping, and form completely different types of connections within the local cortical microcircuitry (Kubota 2014). For example, while PV interneurons provide widespread innervation of pyramidal cells, VIP interneurons appear to predominantly inhibit other interneurons. We found that the MD is preferentially connected with PV interneurons, while the vHPC shows stronger connectivity onto VIP interneurons. While our sample size for the VIP cells is small, especially given their potential heterogeneity (Paul et al. 2017), these results are consistent with retrograde tracing studies demonstrating that both interneuron populations can receive input from the MD and vHPC, but that the vHPC provides stronger innervation of VIP, than PV, interneurons (Sun et al. 2019; Sun et al. 2020). They are also consistent with a recent optogenetics study demonstrating a direct monosynaptic connection between the vHPC and VIP interneurons (Lee et al. 2019). However, functional interpretation of the interneuron effects is complex as our results also suggest that vHPC inputs onto VIP cells would be more likely to be filtered out following repeated stimulation, especially at a higher frequency. Although we have not yet examined the relative connectivity of MD and vHPC inputs onto somatostatin-expressing interneurons, several recent studies suggest that this population may receive input from both structures but be preferentially engaged by vHPC inputs (Abbas et al. 2018; Sun et al. 2019).

### MD and vHPC Inputs Show Differences in their Frequency-Dependent Synaptic Dynamics

In addition to biases in functional connectivity from MD and vHPC inputs onto distinct populations of local interneurons, our results suggest that MD and vHPC inputs can also be distinguished based on frequency-dependent synaptic dynamics. Our dual injection experiments testing the response of MD and vHPC inputs to repeated stimulation at 20 Hz revealed that vHPC inputs showed much greater desensitization in response to repeated stimulations at 20 Hz than MD inputs. As a consequence, the probability of effective synaptic transmission onto pyramidal cells decreased dramatically by the second or third stimulation. Intriguingly, this difference in synaptic efficacy between the two inputs was frequency specific, as stimulation at a lower theta frequency (8 Hz) resulted in much more comparable synaptic dynamics between the two inputs. Importantly, because we are recording from the same cell in response to stimulation of both inputs, the differences in desensitization cannot be attributed to recording from two different postsynaptic neurons. Thus, MD and vHPC inputs onto layer 2/3 pyramidal cells can be distinguished by the most efficient frequencies with which they communicate with the PFC.

These differences in synaptic dynamics could result from either pre- or post-synaptic mechanisms at MD–mPFC versus vHPC–mPFC synapses. Presynaptically, desensitization in response to repeated stimulation has been linked to high baseline release probability or decreases in the number of functional release sites, while postsynaptic changes in the accumulation of desensitized receptors may also contribute (Graziane and Dong 2016). The higher degree of desensitization seen in vHPC relative to the MD inputs at 20 Hz suggests they may initially be higher probability of release synapses, which could reflect differences in various calcium-associated molecules targeted to the presynaptic terminals. Alternatively, vHPC inputs could target postsynaptic sites characterized by glutamate receptors with distinct desensitization kinetics from those targeted by MD inputs. However, prior work mapping the subcellular synaptic connectivity of MD and vHPC inputs to different types of spines on the dendritic arbors of layer 2/3 pyramidal neurons as well as the relative NMDA/AMPA receptor ratios at these synapses suggests that these parameters do not differ (Little and Carter 2012).

It is important to note that the responses observed under our recording conditions likely reflect the contribution of both monosynaptic and polysynaptic inputs. In particular, it is possible that feedforward inhibition initiated via activation of GABAergic interneurons may play a role in dampening down responses of pyramidal cells to repeated stimulations of vHPC inputs at 20 Hz. In the future, this would be an extremely interesting possibility to test by including blockers of GABAergic transmission in the bath.

Interestingly, it appears that desensitization at high frequencies may not be a universal feature of vHPC synapses within the mPFC as vHPC inputs to layer 5 pyramidal cells were shown to sensitize at high frequencies such as 20 Hz (Liu and Carter 2018). This difference might reflect differential targeting of calcium-related machinery to layer 2/3 versus layer 5 presynaptic terminals or differences in glutamate receptor populations found in layer 2/3 versus 5 pyramidal cells. Alternatively, the differences might partially reflect experimental variables, as vHPC sensitization was seen under conditions using a higher extracellular calcium concentration (4 mM) than that used in the current study (2 mM).

The results of our in vitro experiments identifying preferential transmission from the vHPC to layer 2/3 of the mPFC following stimulation at 8 rather than 20 Hz are in line with in vivo experiments suggest that theta frequency oscillations (4–12 Hz) are one of the principal frequencies recorded in the vHPC (Buzsaki 2002). Moreover, vHPC theta appears to be the predominant frequency that modulates PFC firing during anxiety- and working memory-related tasks (Sigurdsson et al. 2010; Padilla-Coreano et al. 2016; Tamura et al. 2017; Padilla-Coreano et al. 2019). In contrast, communication between the MD and the mPFC in the higher beta frequency (20 Hz) was found to predominate during acquisition and performance of the same working memory task (Parnaudeau et al. 2013).

### A High Degree of Convergence between MD and vHPC Inputs Enables Potential Cross-Talk

Our study has focused on how inputs from the MD and vHPC can be distinguished by the mPFC but there may be cross-talk and modulation between the inputs. Some evidence that stimulation of one input might facilitate the other comes from our anecdotal observation that in one case, a functional connection between MD inputs and a VIP cell was revealed following stimulation of vHPC inputs at 8 Hz. A similar type of plasticity has been described in anesthetized mice where stimulation of thalamic inputs to the PFC could either enhance or inhibit subsequent PFC responsiveness to stimulation of hippocampal inputs depending on the frequency and duration of thalamic stimulation and the interstimulus interval (Floresco and Grace 2003). Although not measured, presumably stimulation of hippocampal inputs could also gate PFC responses to thalamic stimulation. These forms of plasticity could be important for regulating PFC-dependent behaviors such as working memory, where MD and vHPC inputs are required during distinct behavioral epochs (Spellman et al. 2015; Bolkan et al. 2017; Abbas et al. 2018). Future studies using the ChrimsonR/Chronos dual stimulation experiments should examine the capacity for MD inputs to modulate vHPC ones, and vice versa.

In sum, we found that MD and vHPC inputs to layer 2/3 pyramidal cells are convergent, but are distinguished by their engagement of different elements of prefrontal interneuron circuitry as well as frequency-specific synaptic dynamics. Although there are many more elements of this connectivity that need to be established, these findings are an important first step in understanding how the prefrontal cortex is capable of both segregating and integrating multiple subcortical excitatory inputs.

## Supplementary Material

Supplementary_Figures_Publication_tgaa084

## Notes

We would like to thank Lindsay Kenney, Caroline Rauffenbart, and Julia Greenwald for their assistance with genotyping mice. Figures created using BioRender.com. The opinions expressed are the authors’ own and do not reflect the view of the National Institute of Mental Health, the National Institutes of Health, the Department of Health and Human Services, or the United States Government. *Conflict of Interest*: The authors declare no conflict of interest.
