# Supplementary material for: Differential Synaptic Dynamics and Circuit Connectivity of Hippocampal and Thalamic Inputs to the Prefrontal Cortex
Source: Cereb Cortex Commun. 2020 Nov 6;1(1):tgaa084. doi: 10.1093/texcom/tgaa084 (PMC7750130; doi:10.1093/texcom/tgaa084)
Supplement: Supplementary_Figures_Publication_tgaa084 [file supplementary_figures_publication_tgaa084.pdf]

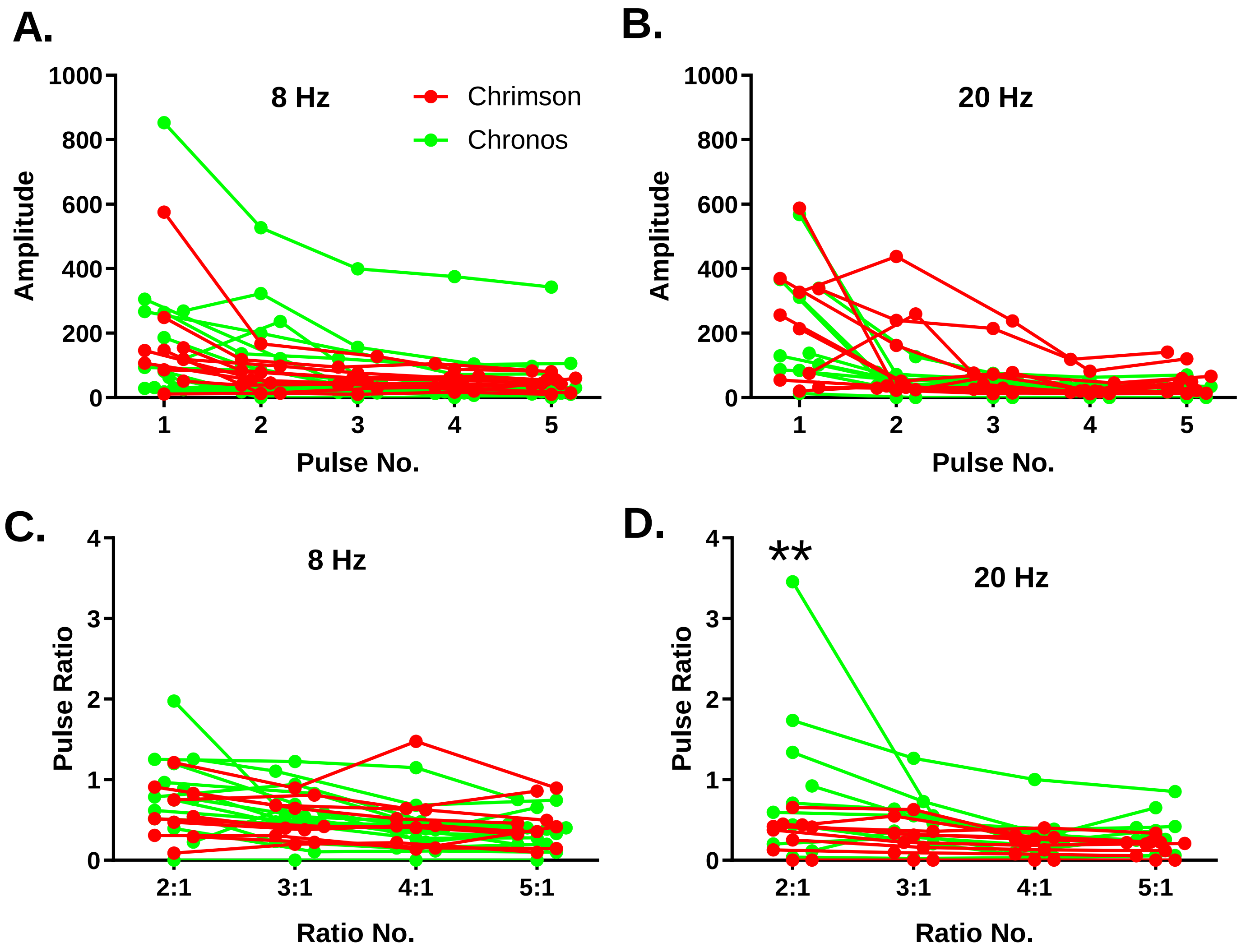

Supplementary Figure 1. Individual data points for cells recorded in Figure 2. (A) The amplitude of individual cell responses to repeated pulses of light at 8 Hz in response to stimulation of MD inputs expressing Chronos (green) or Chrimson (red). **(B)** The amplitude of individual cell responses to repeated pulses of light at 20 Hz in response to stimulation of MD inputs expressing Chronos (green) or Chrimson (red). **(C)** The ratios of individual cell responses to subsequent pulses of light relative to the first pulse in response to stimulation of MD inputs expressing Chronos (green) or Chrimson (red) at 8 Hz . **(D)** The ratios of individual cell responses to subsequent pulses of light relative to the first pulse in response to stimulation of MD inputs expressing Chronos (green) or Chrimson (red) at 20 Hz. **p<0.01.

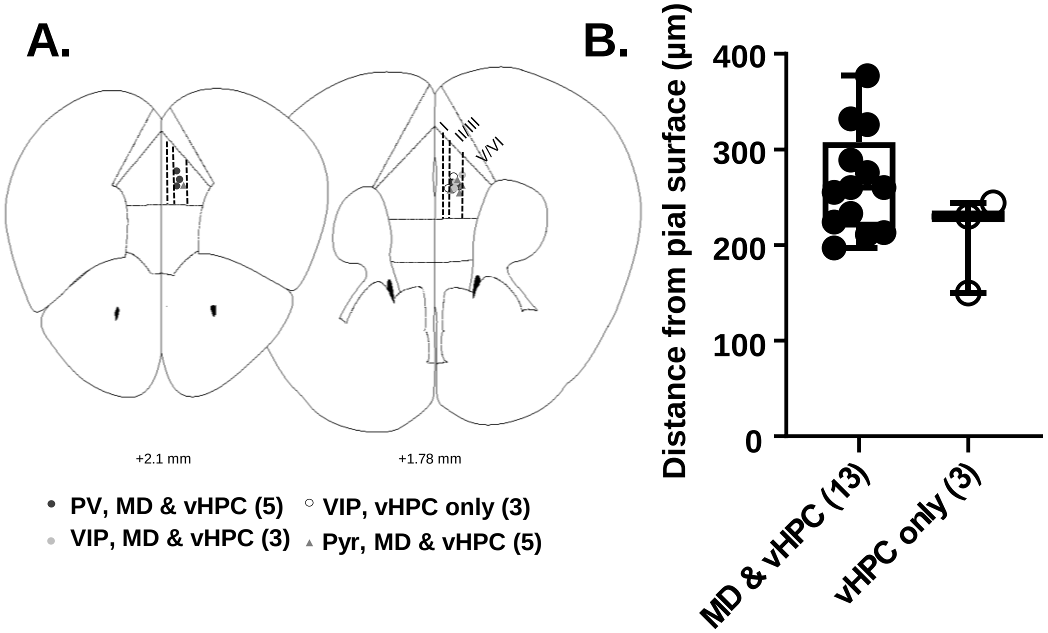

Supplementary Figure 2. Illustration of recording location of all cells where posthoc images were collected. (A) Images confirming the recording location were available for 5 PV, 6 VIP and 5 Pyramidal (Pyr) cells. Locations, generally clustered around the border of layer II and III, are illustrated for PV, VIP and Pyr cells that received input from both the MD and vHPC, as well as VIP cells that responded only to input the vHPC. Cell location was not available for PV cells that responded only to the MD. **(B)** The distance from the pial surface (measured in µm) does not vary between these groups. One-way ANOVA, no effect of region $F(_{2, 13})$=2.345, p=0.135.

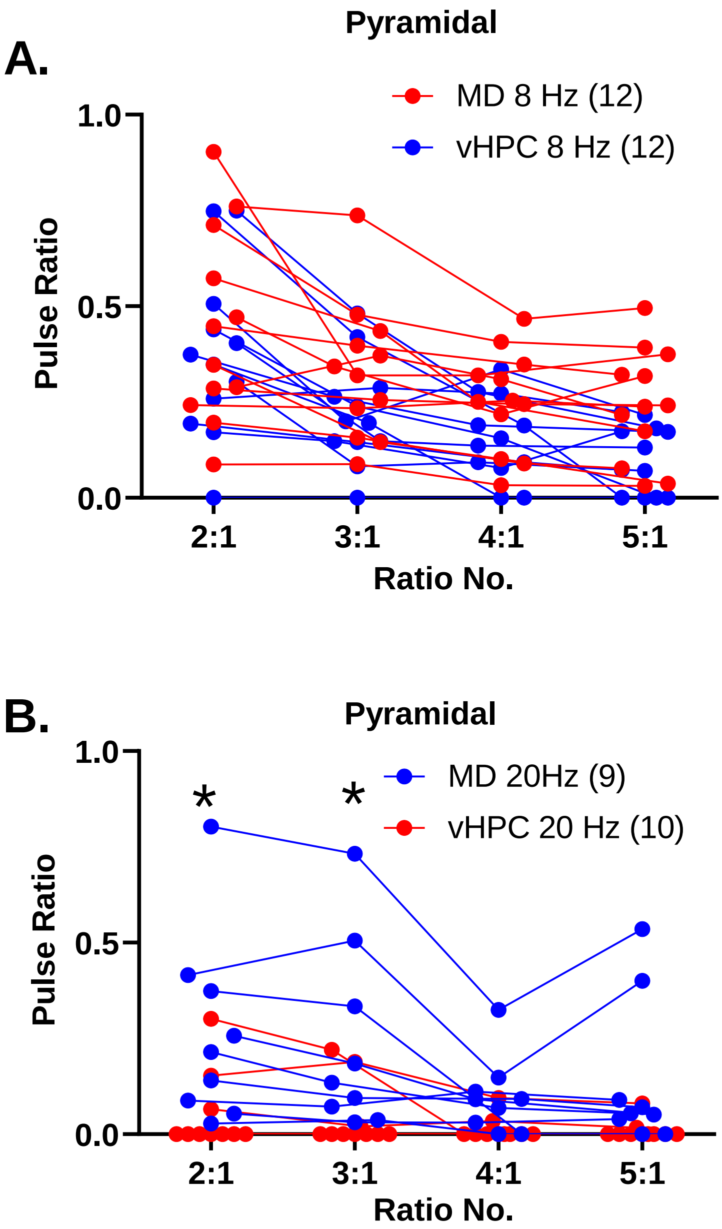

Supplementary Figure 3. Individual data points for cells recorded in Figure 3. (A) The ratios of individual cell responses to subsequent pulses of light relative to the first pulse in response to stimulation of MD inputs (blue) or vHPC inputs (red) at 8 Hz. **(B)** The ratios of individual cell responses to subsequent pulses of light relative to the first pulse in response to stimulation of MD inputs (blue) or vHPC inputs (red) at 20 Hz. *p<0.01.

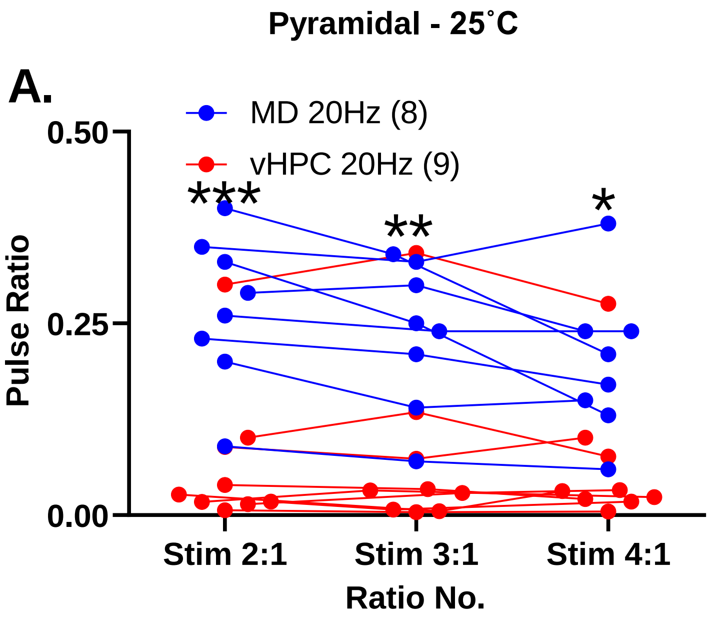

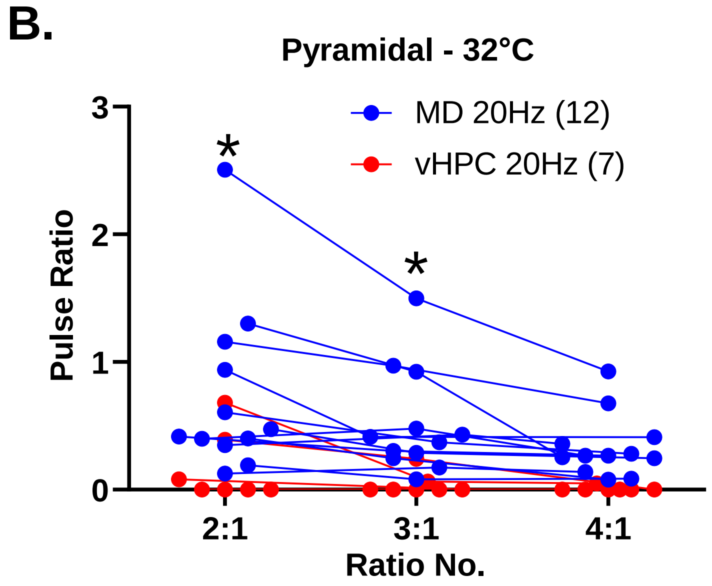

Supplementary Figure 4. Individual data points for cells recorded in Figure 4. (A) The ratios of individual cell responses to subsequent pulses of light relative to the first pulse in response to stimulation of MD inputs (blue) or vHPC inputs (red) at 20 Hz at 25˚C. **(B)** The ratios of individual cell responses to subsequent pulses of light relative to the first pulse in response to stimulation of MD inputs (blue) or vHPC inputs (red) at 20 Hz at 32˚C. *$p<0.05$. **$p<0.01$. ***$p<0.001$.

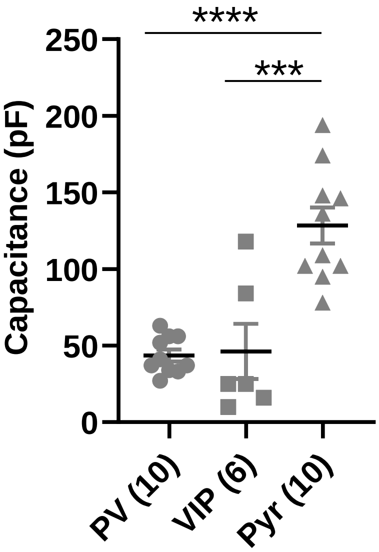

Supplementary Figure 5. Interneuron capacitance is significantly different from that of pyramidal cells. Capacitance measurements for PV and VIP interneurons were significantly smaller than those for Pyramidal cells (Pyr). One-way ANOVA, $F_{(2, 23)}=21.06$, $p<0.0001$. Bonferroni post-hoc PV v Pyr, multiplicity-adjusted ****$p<0.0001$; VIP v Pyr, multiplicity-adjusted ***$p=0.0001$.

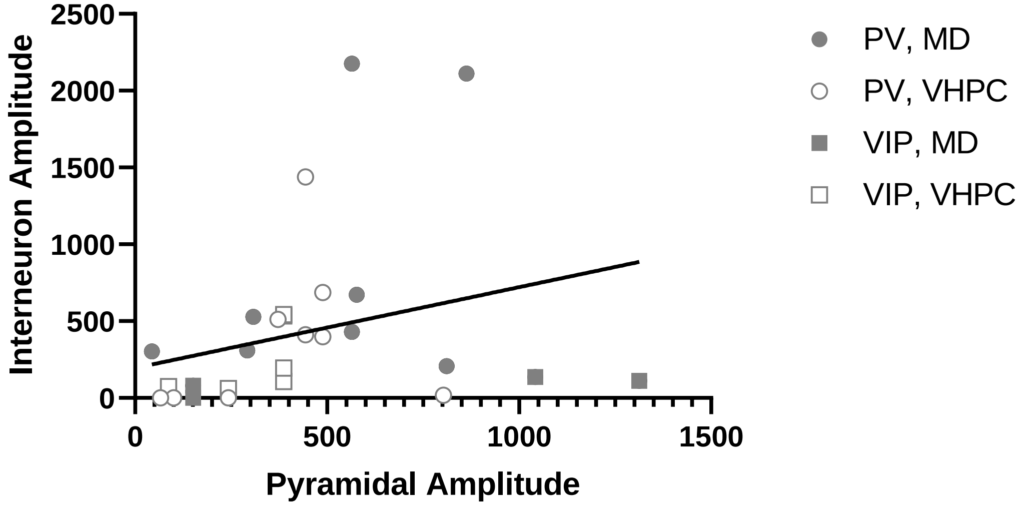

Supplementary Figure 6. Interneuron response amplitude is not significantly correlated with Pyramidal response amplitude. The amplitude of each interneuron response is plotted against the corresponding amplitude of the Pyramidal cell recorded nearby. The best fit line describing the relationship between these two variables is plotted in black but the slope of this line in not significantly different from zero. Linear regression, $F_{(1, 28)}$=2.55, p=0.12.

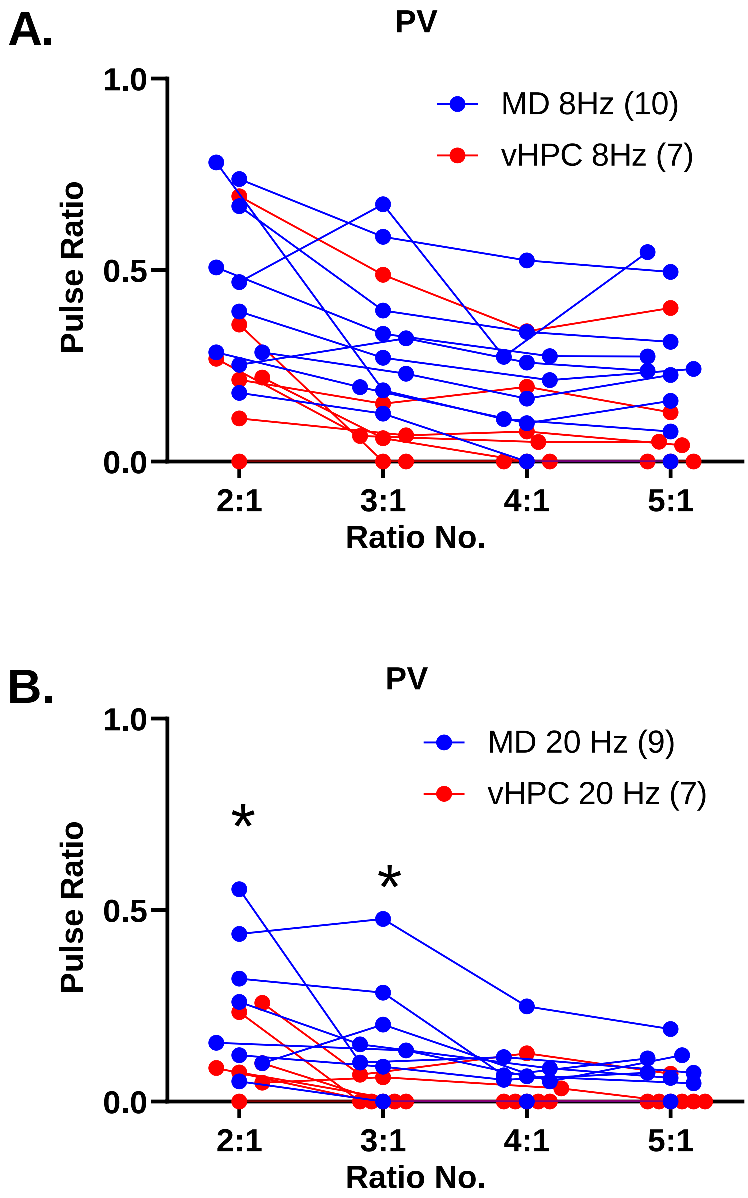

Supplementary Figure 7. Individual data points for cells recorded in Figure 5. (A) The ratios of individual cell responses to subsequent pulses of light relative to the first pulse in response to stimulation of MD inputs (blue) or vHPC inputs (red) at 8 Hz. **(B)** The ratios of individual cell responses to subsequent pulses of light relative to the first pulse in response to stimulation of MD inputs (blue) or vHPC inputs (red) at 20 Hz. *$p<0.05$.

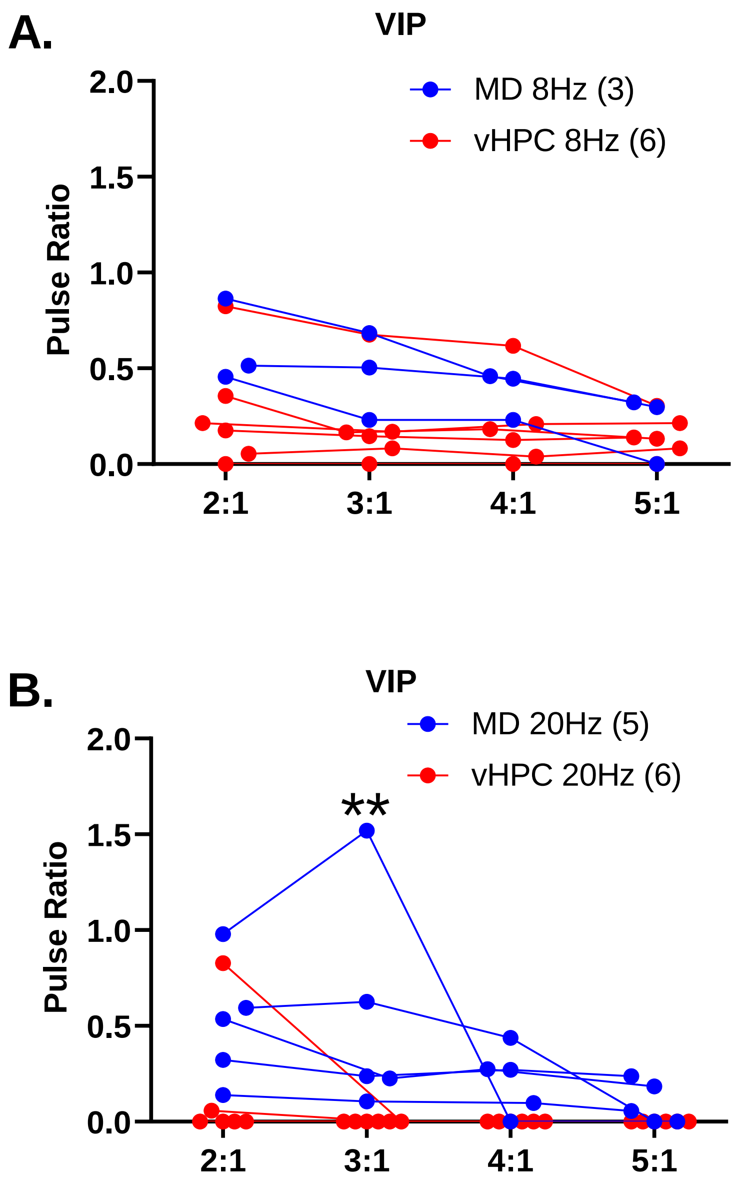

Supplementary Figure 8. Individual data points for cells recorded in Figure 6. (A) The ratios of individual cell responses to subsequent pulses of light relative to the first pulse in response to stimulation of MD inputs (blue) or vHPC inputs (red) at 8 Hz. **(B)** The ratios of individual cell responses to subsequent pulses of light relative to the first pulse in response to stimulation of MD inputs (blue) or vHPC inputs (red) at 20 Hz. **$p<0.01$.
